# Advancing the community plan to end the HIV Epidemic in Philadelphia: a qualitative descriptive evaluation of low-threshold PrEP services in sexual health clinics

**DOI:** 10.1186/s43058-023-00543-y

**Published:** 2024-01-05

**Authors:** Stephen Bonett, Anjali Mahajan, Daniel Teixeira da Silva, Javontae Williams, Kathleen Brady, José Bauermeister, Sarah M. Wood

**Affiliations:** 1 School of Nursing, University of Pennsylvania, Philadelphia, PA USA; 2 The University of Oxford, Oxford, England; 3 Philadelphia Department of Public Health, Philadelphia, PA USA; 4 Perelman School of Medicine, University of Pennsylvania, Philadelphia, PA USA; 5 Craig Dalsimer Division of Adolescent Medicine, Children’s Hospital of Philadelphia, Philadelphia, PA USA

**Keywords:** Pre-exposure prophylaxis, HIV prevention, Consolidated Framework for Implementation Science, Implementation strategies, Sexual health

## Abstract

**Background:**

Pre-exposure prophylaxis (PrEP) is an effective HIV prevention method and a key component of Philadelphia’s Community Plan to End the HIV Epidemic (EHE). However, significant barriers to accessing PrEP exist among people at risk for HIV. Low-threshold models for PrEP services that minimize barriers to entry and service engagement could help bolster access to PrEP through community-based clinics. This study aimed to describe the initial implementation of low-threshold PrEP services in three sexual health clinics funded by the Philadelphia Department of Public Health and explore strategies for delivering low-threshold PrEP services.

**Methods:**

We conducted three focus groups with staff (i.e., providers, prevention navigators, and administrative staff, *N* = 21) at each of three participating PDPH-funded sexual health clinics from November 2021 to January 2022. Discussion topics included details about the PrEP delivery process, clinic strengths and assets, resource gaps, and PrEP implementation goals. Follow-up interviews with staff members (*N* = 8) between March 2022 and May 2022 focused on identifying successful strategies for PrEP delivery and adaptations needed to optimize low-threshold PrEP service delivery. Rapid qualitative methods and the Consolidated Framework for Implementation Science were used to analyze data from focus groups and interviews.

**Results:**

Participants collaborated to create process maps that visualized the steps involved in delivering PrEP services within their respective settings. These maps highlighted several stages in PrEP service delivery, such as connecting individuals to services, providing prevention navigation, conducting clinical encounters, and ensuring follow-up care. Participants described effective strategies for implementing PrEP, which included integrating and co-locating services on-site, strengthening staffing resources and capacity, and addressing barriers experienced by clients.

**Conclusions:**

Lessons from the implementation of low-threshold PrEP service delivery in Philadelphia can guide ongoing local adaptations and future scale-up of these models to improve access to PrEP and advance the goals of the EHE initiative.

**Supplementary Information:**

The online version contains supplementary material available at 10.1186/s43058-023-00543-y.

Contributions to the literature

•“Low-threshold” PrEP services are streamlined, patient-centered models of PrEP delivery that can facilitate access to care by lowering systems-level barriers
•We conducted focus groups and interviews with staff at sexual health clinics funded to deliver low-threshold PrEP services. We used rapid qualitative analysis to describe these low-threshold models, identify implementation strategies, and explore factors that could facilitate or hinder their use.
•Our paper contributes to the literature by using implementation science to characterize approaches to low-threshold PrEP delivery in sexual health clinics funded by a local health department as part of national efforts to End the HIV Epidemic.

## Introduction

In 2019, Philadelphia was prioritized for the Ending the HIV Epidemic in the US (EHE) initiative, aiming to scale up HIV prevention and treatment strategies and reduce new infections by 90% by 2030 [1]. Pre-exposure prophylaxis (PrEP) is a safe and effective prevention method for those at high risk of HIV [2]. Despite increasing awareness, PrEP uptake remains low in Philadelphia and across the country [3, 4]. Importantly, Black and Latinx individuals access PrEP less than White individuals, widening racial disparities in HIV burden [5–7].

Previous studies have identified systems-level barriers to PrEP uptake, including insufficient clinical navigation support [8, 9], cost and insurance concerns [10], limited access to PrEP providers [11], and discrimination in healthcare settings [12]. Structural barriers like transportation, housing, and employment also impact access [13]. Streamlined, integrated models of PrEP service delivery can reduce these barriers [14–16]. For example, patient navigation systems (i.e., assisting patients in overcoming barriers and providing psychosocial support) have shown benefits [17, 18]. These models, referred to as “low-threshold” PrEP delivery, employ strategies to enhance systems-level access and are inspired by harm reduction approaches [19].

To address ongoing PrEP barriers, the Philadelphia Department of Public Health (PDPH) developed a Community Plan to End the HIV Epidemic, focusing on HIV diagnosis, treatment, and prevention, including expanding PrEP access [20]. The plan aims to create accessible PrEP initiation models that tackle systems-level barriers. In 2021, the city funded sexual health clinics to offer low-threshold access to services, such as HIV and STI testing, rapid linkage to PrEP and HIV treatment, on-site PrEP and HIV treatment, and patient navigation. This service expansion offers a unique opportunity to evaluate the implementation of low-threshold models of PrEP care at the clinic level and gain key insights into their impact and scalability.

Given the need to better characterize models for providing low-threshold PrEP services, we aimed to describe the initial implementation of low-threshold PrEP services in three PDPH-funded sexual health clinics and explore strategies for delivering low-threshold PrEP services.

## Methods

This cross-sectional qualitative descriptive study recruited staff from three sexual health clinics in Philadelphia, PA, USA, for focus groups and interviews. These clinics received PDPH funding for planning and implementing low-threshold sexual health services over a 5-year period starting in 2021. The grant required clinics to offer HIV and STI testing, rapid treatment linkage, pregnancy testing, PrEP services, non-occupational post-exposure prophylaxis (nPEP) services, and patient navigation. The clinics funded through this grant had implemented components of the model to various degrees, but none had adopted the full suite of funding-specific services and principles. Four clinics were funded, and three participated in the study; one clinic declined due to staffing and leadership transitions. Institutional Review Board approval was obtained from the City of Philadelphia and the University of Pennsylvania. The study followed the Standards for Reporting Qualitative Research guidelines (Supplemental file 1). Additional details about the focus group methodology, interview methodology, and the rapid qualitative analysis can be found in Supplemental file 2.

### Focus groups

We completed focus groups with each of the three sexual health clinics between November 2021 and January 2022, with a public health nurse researcher (SB) facilitating each focus group. Focus groups lasted approximately 60 min and included a process mapping activity using swim lane diagrams [21]. A total of 21 participants participated in the three focus groups. One participant had previously been interviewed for another study by the researcher facilitating the focus groups; no other participants had a prior relationship with the focus group facilitator. Audio from the focus groups was recorded and transcribed verbatim by a third-party professional transcription company. Written informed consent was provided by all focus group participants prior to the start of the focus group.

### Interviews

Leadership at each clinic was asked to distribute information about participating in these interviews to their staff. In-depth interviews were conducted by a public health nurse researcher (SB) with eight key stakeholders across the three clinics between March and May 2022. Participants completed a 45–60-min semi-structured interview, guided by the Consolidated Framework of Implementation Science (CFIR), over a video conferencing platform [22]. Of the eight participants who completed an interview, five had participated in the previous focus groups. Audio from the interviews was recorded and transcribed verbatim by a third-party professional transcription company, and no additional fieldnotes were recorded. Written informed consent was provided by all participants prior to the interview.

### Rapid qualitative analysis

All potentially identifiable information, including names and specific job titles, was removed from transcripts by the research assistant (AM) prior to analysis to protect participant anonymity. Rapid qualitative analysis was conducted using transcript summaries and matrix analysis [23–25]. Summary templates were developed for the focus groups with domains corresponding to the main sections of the focus group guide (i.e., process mapping, strengths, challenges, and goals). Templates were developed for the interviews with domains corresponding to questions from the semi-structured interview guide and mapping to CFIR domains and constructs. Analytic matrices were created using these summary templates. Analysts then proceeded with both row-wise and column-wise analysis to explore themes arising within specific transcripts and themes arising by domain across multiple transcripts. The findings that emerged from the rapid qualitative analysis were presented to and discussed with each of the participating clinics and to the Philadelphia Department of Public Health in July–September, 2022.

## Results

During focus groups and interviews, participants identified 17 distinct strategies for lowering barriers to PrEP access in their settings. These strategies were mapped onto 11 strategies from the Expert Recommendations for Implementing Change (ERIC) compilation [26] and were grouped into six thematic groups (Table 1). Additional results from the process mapping component of the focus groups and findings related to implementation determinants can be found in Supplemental file 3.

**Table 1:** Strategies for lowering barriers to PrEP access identified in interviews and exemplar quotes

**Themes**	**ERIC implementation strategies**	**Participant identified strategies**	**Quote**
*Conducting Community Outreach*	Prepare patients to be active participants	In-person outreach, especially for young adults	“Even at college events, I feel like students are more comfortable at those events. They’re like, ‘Hey, I actually never heard of PrEP,’ and then they’re like, ‘Oh, I’m comfortable in this setting,’ rather than being inside an office or, like, in a lab room, talking about it.” (Prevention Navigator)
*Building External Collaborations*	Develop academic partnerships	Leveraging resources and education from pharmaceutical representatives, researchers, and clinician partners to enhance PrEP awareness.	“A very consistent strategy that we’ve been using is collaboration with pharmaceutical companies that provide PrEP... They’ve not only been offering these presentations for the staff. They’ve been offering them for clients as well... and I know we collaborate with [local healthcare system] and their research team a lot, but we need to start collaborating with them on the level of them educating the clients or showing them what they found through their research” (Prevention Navigator)
	Promote network weaving (external)	Collaboration with other community organizations to leverage collective resources.	“Sometimes it’s nice to collab. If one org has something that can help this community in another way, and then we can help related to testing in HIV and PrEP.” (Prevention Navigator)
*Providing Co-located Resources and Services*	Change service sites	One-stop-shop for services	“Not just sexual health, but they have education. They have a college here. They have other resources. They have a GED program. They have CDL. You know, there’s so much other things that [agency name] had refocused, I believe, throughout the years to make sure that they’re client-centered.” (Prevention Navigator)
		In-house pharmacy and using home-delivery services through outside pharmacies	“At the end of the day, it’s not like a pharmacy when you go to Rite Aid, and they’ll be like, ‘Hi. Okay. You’re done.’ No. They’re, like, ‘Oh, hola, [removed]. How are you?’ You know? They interact. They get to know you because these people have been coming, getting their prescription for years. So they feel at home within the pharmacy that’s inside [agency name].” (Prevention Navigator)
		Providing services from a Mobile Treatment Unit.	“We’re not the only people with mobile vans, there’s 10 of us now in Philadelphia... It’s just a helpful thing to get us to the people are the most vulnerable and underserved.” (Agency Leader)
	Promote network weaving (internal)	Close partnership between prevention team and onsite clinic	“Because us, we’re here, and when we get people connected-since our clinic is right next door- you don’t have to travel down the street, or you don’t have to catch another bus or anything. You could get the insurance from us and then if you have any questions, we’ll answer it with you when we’re signing up. And then whenever you get approved, you don’t even have to come to [the prevention team] anymore. You can just go to the clinic. Or, if you wanna do a same-day appointment, we just call the clinic and we can walk you over.” (Prevention Navigator)
*Integrating PrEP into Clinical Services*	Revise professional roles	Using an education specialist to provide in-depth information about PrEP for individuals and groups	“Now we have funding for this [education specialist], which makes it better. Because we always said that this was the missing component because, we did educate them but we couldn’t document it. You know, we could document in our reports but we couldn’t go into depth about what was discussed and so forth like that. Now on the other hand it’s different.” (Prevention Navigator)
		Addressing social needs through integrative medicine	“I think this is the problem in scaling up PrEP... if you peel it off and give it to some peripheral person, most of the clinicians aren’t talking about it, thinking about it, engaged in it, engaged with the patients and dealing with it, helping the patients deal with it. See how it inter-relates to other things. So integrative medicine is what we do and what we’re here for... I think it is key. (Agency Leader)
	Remind clinicians	PrEP integrated into forms and protocols, prompting conversation.	“I think integrating [PrEP into the intake paperwork] has been helpful, because it gives them a chance to talk about it, and you have to explain what it is, because I think a lot times we notice that people were just saying, ‘Would you like to start PrEP?’ And the patient would say, ‘No’ but there was no like education on what it was.” (Prevention Manager)
*Increasing Staffing Resources and Capacity*	Recruit, designate, and train for leadership	Hiring staff from within the communities being served	“I think [agency name] actually does a great job of when they’re interviewing folks of trying to find folks that are part of the community that we are trying to serve. So, instead of trying to reinvent the wheel, if we’re looking to reach out to MSM and trans folks, not hiring a cis person to work directly with those populations.”(Prevention Navigator)
	Facilitate relay of clinical data to providers	Reporting metrics around PrEP goals to team	“We have monthly staff meetings so that’s how we get updated, I mean, ‘cause we’re the testers but we can’t always know exactly the numbers. So, usually our supervisors will, after we give them our report, they’ll put it into their report to submit and then when we have a staff meeting, we report back to everybody like on a monthly basis how we’re doing.” (Prevention Navigator)
*Addressing Client-Level Barriers*	Intervene with patients to enhance uptake and adherence	Motivational interviewing	“We believe that motivational interviewing is a key strategy that really everyone who has any role in patient care should be using... there’s exponentially more research supporting motivational interviewing than any other psychosocial intervention anywhere, right? And so if there’s one thing we should all be focused on, it’s this.” (Agency Leader)
		Linking uninsured or undocumented clients to patient assistance programs.	“Then it’ll come up, ‘Well like, I don’t have insurance,’ or, ‘Is it a copay involved?’ Like, ‘How am I gonna pay for this?’ Right? And it’s something like, ‘Oh, don’t worry about it. We’ll cover it. Like, it will be nothing to you.’... not only do we have that assistance program that covers the PrEP medication and the PrEP appointments, this particular grant has some money that will also cover labs.” (Prevention Manager)
		Opt-out PrEP services.	“…we have been starting to implement kind of an opt-out rather than an opt-in process. So, when we do get a new patient, we kind of say that as of a blanket, for all of our patients we do rapid HIV bloodwork and a urine STI screening. Which they can decline if they want to, but, usually they don’t. And then we can talk to them about the services, like PrEP and that kind of stuff, and have them decline getting it rather than saying ‘Oh, yeah, I do want that.’” (Prevention Manager)
		Offering PrEP information to all clients.	“We’re not known for testing like just LGBT people, we’re not known for just testing women, we’re not known for just testing men. We test anybody who walks through the door for everything. I think that’s a great benefit in the sense of we’re not telling people anymore that, ‘Oh no, we’re just trying to get MSM or trans folks on PrEP.’” (Prevention Navigator)
		Incentives for HIV testing and PrEP maintenance (client-level)	“The current clients, they come back for their gift cards and their key passes, and then they get their meds. So, they’re going to remember, like, ‘Oh, if I don’t get my meds, then I won’t get this.’” (Prevention Navigator)

### Key implementation strategies

#### Conducting community outreach

Community outreach strategies were identified as important for reaching key populations, including young adults and students. These strategies included health fair participation, tabling events in community settings, and promoting peer-to-peer education. Clinics cited meeting people outside of the clinical setting as a way to lower initial barriers to service engagement.

#### Building external collaborations

Participants identified building external collaborations as an important strategy for leveraging resources and enhancing PrEP awareness. Potential collaborators included pharmaceutical representatives, academic researchers, clinical partners, and other community organizations. It was also noted that strong communication among different community organizations was essential for building mutually beneficial relationships and referral systems.

#### Providing co-located resources and services

Co-locating social and medical services was seen as crucial to lowering access barriers and providing holistic sexual wellness care. In line with guidance from the PDPH funding, all clinics adopted variations on a “one-stop shop model” for sexual wellness care. These models integrated HIV and STI prevention navigation and clinical care, as well as a wide range of social services including assistance acquiring identification documents, harm-reduction supplies for injection drug use, GED programs, and food and clothing pantries. Warm hand-offs between prevention and clinical teams and systems for scheduling same-day appointments were viewed as particularly effective in this context.

#### Integrating PrEP into clinical services

Participants described several strategies for integrating PrEP services into the clinics’ broader clinical care and service delivery models. Two organizations adopted strategies to “nudge” staff and clinicians to address PrEP within clinical encounters, by adding questions about PrEP to patient intake forms. These questions served as prompts to address PrEP during visits, facilitate in-house referrals, and meet client-specific needs. One organization highlighted that having medical providers address a broad range of medical and social needs during clinical encounters, beyond sexual health concerns, served to integrate sexual health into a holistic care model and could lower barriers to care across health services.

#### Increasing staffing resources and capacity

Across sites, participants discussed how hiring staff with experiences and identities reflecting the communities served by their clinics was a key strategy to increase engagement with PrEP services. Participants highlighted how bolstering staff recruitment efforts from Black, Latinx, queer, transgender, and immigrant communities would strengthen the clinics’ cultural connection to clients, though clinics varied in the degree to which they had implemented these efforts. One participant suggested that expanding access to training opportunities and enhancing wages and benefits would promote the recruitment and retention of staff with lived experience and community expertise.

#### Addressing client-level barriers

Participants described a variety of strategies aimed at addressing client-level barriers to PrEP access, ranging from increasing awareness and soliciting client motivations for using PrEP to connecting clients to patient assistance programs. Staff at all three organizations reported offering PrEP counseling universally to clients and incorporated PrEP into broader conversations of sexual wellness. This approach to PrEP counseling was perceived to facilitate client-centered and non-stigmatizing PrEP access and ensure that all clients were knowledgeable about PrEP. Participants identified cost and insurance as key barriers and discussed how efforts to connect their clients to patient assistance programs, enroll in public insurance, and navigate existing insurance coverage were fundamental for connecting clients to PrEP services.

## Discussion

In this study, staff at newly funded sexual health clinics in Philadelphia described their low-threshold PrEP care delivery models and implementation strategies. In total, 17 distinct strategies were identified, and four strategies were consistently identified at all three sites as cross-cutting strategies with the potential to improve PrEP access for marginalized populations. These key strategies were (1) co-locating services in a one-stop-shop model (e.g., HIV/STI testing and treatment, PrEP and PEP services, patient navigation, social services), (2) integrating universal PrEP counseling and expanding efforts to build trust with communities through, (3) conducting community outreach, and (4) diversifying the clinical workforce in alignment with the communities being served.

Co-locating and integrating services into a “one-stop shop model” was a key strategy for lowering barriers to accessing PrEP for the study clinics. Having clinical PrEP services co-located with prevention navigation services was particularly beneficial for maintaining engagement with clients and capitalizing on PrEP readiness by providing a frictionless path to meet with a PrEP provider. This finding aligns with previous work suggesting that on-site referrals to PrEP can help to reduce the number of clients who express PrEP interest during navigation but do not link to care following a referral to an outside organization [27]. Integration of PrEP services with other social services was also highlighted as a promising strategy. Care delivery models incorporating integrated social services have been particularly effective in lowering access barriers for individuals facing social and economic marginalization [16]. Recent research studying PrEP service integration with syringe service and substance use treatment programs suggests that these co-located models could have significant benefits for promoting client engagement and retention [28–31]. Co-locating and integrating prevention and clinical PrEP services also has scheduling benefits. Long PrEP appointment wait times have been identified as a barrier to uptake and a juncture where many clients fall off the PrEP care continuum [11, 27]. On-site clinical PrEP services facilitate rapid and flexible PrEP appointment scheduling, often allowing for same-day appointments. In our study, all three sites had same-day scheduling, with two sites using it as the primary process for new clients. Other studies have found that the ability to schedule same day appointments for PrEP services enhanced PrEP uptake [32–34].

Participants in this study identified universal PrEP counseling as another key implementation strategy for increasing PrEP access. Participants described how discussing PrEP with every client helped normalize conversations about PrEP. Researchers and clinicians have called for greater efforts to routinize PrEP counseling across healthcare settings including primary care and family planning clinics [35, 36]. By integrating frequent and consistent PrEP discussions into clinical practice, healthcare providers can help to link people to PrEP care in moments of high need and motivation [37]. Universal PrEP counseling can also help avoid missing potential candidates who are not comfortable asking about PrEP or disclosing key risk factors during risk-based screening and is aligned with a shared decision-making framework where patients and providers work together to make decisions about health and wellbeing [35, 38]. In their latest clinical practice guidelines for PrEP, the Centers for Disease Control have included a recommendation to discuss PrEP with all sexually active people [39].

Our study participants discussed a variety of strategies to build sustained, trusting relationships with the communities they served. These strategies ranged from outreach efforts to maintain a consistent community presence to organizational efforts to hire staff with lived experiences that resonate with their clients and the community. A key determinant of the success of these strategies was having an organizational culture that fostered affirming environments for people of diverse identities. These insights align with other studies examining staff and provider perspectives on PrEP care models. In studies of clinical and non-clinical PrEP service providers, those who shared aspects of their identity with their clients, or who had personal experience using PrEP, felt better able to connect with and support their clients [38, 40]. Importantly, researchers have highlighted the role of recruiting staff and leaders from the specific communities they serve as a key strategy for mitigating stigma and building long-lasting community integration [41].

Notably, the strategies described by participants for lowering systems-level barriers to accessing PrEP are largely in alignment with the model provided by the PDPH grant funding these clinics. The integration of multiple medical and social services onsite, adoption of universal PrEP counseling, and prioritization of same-day scheduling are key aspects of the health department’s model and were identified by participating staff as successful strategies on the ground. This alignment is an indicator that staff in these community-based clinics have shared ownership of these low-threshold PrEP delivery models. Achieving buy-in from implementation partners can help support the long-term integration and sustainability of these service delivery models in community settings.

This study has notable limitations. We examined three sexual health clinics in Philadelphia that received support from the city’s health department for implementing low-threshold sexual health services. While valuable for understanding local implementation within an Ending the HIV Epidemic plan, the findings may not apply to other local contexts or HIV service providers in Philadelphia not funded under this initiative. Additionally, while this study provides insightful contextual information on early implementation, data on implementation and client outcomes are lacking. Future research should investigate the impact of low-threshold PrEP care models on outcomes such as reach to priority populations, clinic time and costs, service effectiveness and equity, and PrEP uptake and adherence. Moreover, this study focused solely on staff perspectives and did not include input from clients or potential clients. Further research should explore how individuals, particularly from historically marginalized communities, perceive low-threshold service delivery models for PrEP.

## Conclusion

To achieve the goals of the Ending the HIV Epidemic in the United States initiative, systems-level adaptations are needed to lower barriers to PrEP access and expand access to Black, Latinx, and other historically marginalized communities. Local health departments play a vital role in this effort and can provide both funding and long-term planning that supports community efforts to strengthen access to HIV prevention tools. The strategies described in this study can be adapted by other Ending the HIV Epidemic jurisdictions and tailored to their local context to support the expansion of PrEP access.

### Supplementary Information

**Additional file 1: Supplemental file 1.** Standards for Reporting Qualitative Research reporting guidelines.

**Additional file 2: Supplemental file 2. **Additional Methods Details.

**Additional file 3: Supplemental file 3.** Additional Results Details.

## Data Availability

The data used in this research (which includes transcripts from in-depth interviews and focus groups) is not publicly available due to confidentiality policies.

## Abbreviations

PrEP: Pre-exposure prophylaxis
EHE: Ending the HIV Epidemic in the US
CFIR: Consolidated Framework for Implementation Research
PDPH: Philadelphia Department of Public Health
ERIC: Expert Recommendations for Implementing Change
nPEP: Non-occupational post-exposure prophylaxis
