# Supplementary material for: Advancing the community plan to end the HIV Epidemic in Philadelphia: a qualitative descriptive evaluation of low-threshold PrEP services in sexual health clinics
Source: Implement Sci Commun. 2024 Jan 5;5:4. doi: 10.1186/s43058-023-00543-y (PMC10768374; doi:10.1186/s43058-023-00543-y)
Supplement: Supplementary file 1 — Additional file 1: Supplemental file 1. Standards for Reporting Qualitative Research reporting guidelines. [file 43058_2023_543_MOESM1_ESM.doc]

Supplemental File 1

**Standards for Reporting Qualitative Research (SRQR)**

| **No. Topic** | **Section** |
| --- | --- |
| **Title and abstract** |  |
| S1 Title | Title page |
| S2 Abstract | Abstract |
| **Introduction** |  |
| S3 Problem formulation | Introduction, paragraphs 1-3 |
| S4 Purpose or research question | Introduction, paragraph 4 |
| **Methods** |  |
| S5 Qualitative approach and research paradigm | Methods, paragraph 1; Methods: Rapid Qualitative Analysis; and Supplemental File 2 (Additional Methods Details) |
| S6 Researcher characteristics and reflexivity | Methods: Focus Groups and Methods: Interviews |
| S7 Context | Methods, paragraph 1 |
| S8 Sampling strategy | Methods, paragraph 1 and Methods: Interviews |
| S9 Ethical issues pertaining to human subjects | Methods, paragraph 1 |
| S10 Data collection methods | Methods: Focus Groups; Methods: Interviews; and Supplemental File 2 (Additional Methods Details) |
| S11 Data collection instruments and technologies | Supplemental File 2 (Additional Methods Details) |
| S12 Units of study | Methods: Focus Groups and Methods: Interviews |
| S13 Data processing | Methods: Rapid Qualitative Analysis and Supplemental File 2 (Additional Methods Details) |
| S14 Data analysis | Methods: Rapid Qualitative Analysis and Supplemental File 2 (Additional Methods Details) |
| S15 Techniques to enhance trustworthiness | Methods: Rapid Qualitative Analysis and Supplemental File 2 (Additional Methods Details) |
| **Results/Findings** |  |
| S16 Synthesis and interpretation | Results |
| S17 Links to empirical data | Table 1, exemplar quotes |
| **Discussion** |  |
| S18 Integration with prior work, implications, transferability, and contribution(s) to the field | Discussion, paragraphs 1-5 |
| S19 Limitations | Discussion, paragraph 6 |
| **Other** |  |
| S20 Conflicts of interest | Competing interests statement |
| S21 Funding | Funding statement |

O’Brien B.C., Harris, I.B., Beckman, T.J., Reed, D.A., & Cook, D.A. (2014). Standards for reporting qualitative research: a synthesis of recommendations. *Academic Medicine, 89(9)*, 1245-1251.
