# Supplementary material for: Advancing the community plan to end the HIV Epidemic in Philadelphia: a qualitative descriptive evaluation of low-threshold PrEP services in sexual health clinics
Source: Implement Sci Commun. 2024 Jan 5;5:4. doi: 10.1186/s43058-023-00543-y (PMC10768374; doi:10.1186/s43058-023-00543-y)
Supplement: Supplementary file 2 — Additional file 2: Supplemental file 2. Additional Methods Details. [file 43058_2023_543_MOESM2_ESM.docx]

**Additional Methods Details**

**Model for Low-Threshold Sexual Health Service Delivery**

This grant outlined a sexual health service delivery strategy that was based on a successful model implemented at the city’s Health Center #1, a public health clinic focusing on STD testing and treatment. This model is centered around three core objectives: 1) community engagement, 2) low-threshold access, 3) comprehensive sexual health services. Low-threshold access was defined as care delivery models that provided easy access to services in a customer service centered environment, incorporated a harm reduction approach to sexual health, provided services beyond regular business hours (i.e., early, late, and weekend hours), integrated multiple services on site, facilitated rapid linkage to care, and served clients regardless of insurance status.

**Focus Groups**

A focus group guide was developed and pilot-tested with a group of physicians, research coordinators, and research assistants with expertise in community-based HIV care (see Process Mapping Focus Group Facilitation Guide below). Process mapping was used to compile the knowledge and experience of staff members involved with PrEP delivery into a schematic of the steps taken for a new client to receive a PrEP prescription and/or medication. The schematic developed during the focus group was revised and reformatted for clarity by the study team after the focus group concluded. The completed process map for each center was organized around tasks and responsibilities across departments and staff using a swim-lane model, a validated method for modeling healthcare processes often used in the diagnostic phase of quality improvement efforts.(1) A de-identified example can be found in below. The process map and other notes created during the focus group were adapted by the study team to create diagrams and summaries of each clinic’s process which were shared with the participating clinics within one month of the date of the focus group.

**Interviews**

A semi-structured interview guide (see Interview Guide below) was developed based on the Consolidated Framework for Implementation Research (CFIR) that included questions that assess key domains from this framework (i.e., characteristics of the intervention, process, outer setting, and inner setting).(2) During the interviews, participants were first asked to reflect on successful implementation strategies (i.e., defined efforts and approaches to promote and sustain the adoption of low-threshold PrEP delivery) their clinics were using to lower barriers to accessing PrEP. For each strategy described by the participant, the researcher asked a series of follow-up questions about characteristics of that specific strategy (e.g., “How does this strategy for making PrEP easy to access compare with previous strategies or alternative strategies?”; ”Are there components of this strategy that should be altered?”; “Are there components of this strategy that should NOT be altered?”). Additional questions were then asked about the process, outer setting, and inner setting domains. Before discussing the “process” domain, participants were given several minutes to review the process map that was produced during the focus group at their clinic. Subsequently, questions regarding changes, modifications, or adaptations to the PrEP delivery process were asked in reference to this shared understanding of the delivery process at their clinic.

**Rapid Qualitative Analysis**

Two members of the research team (SB and AM) used the summary template created for the focus groups to independently create summaries of each focus group transcript, then met to integrate the two versions into a single final summary. An analytic matrix was created to visualize the summaries across the three focus groups and was used by the two researchers to complete a rapid analysis of the focus groups.

Two members of the research team (SB and AM) piloted the summary template for the interviews with two transcripts to develop a consistent method for completing the summaries and adjusted the template as needed. The remaining six interview transcripts were split between the two researchers who created final summaries. Two analytic matrices were created from these interview transcript summaries. One matrix was used to visualize the key implementation strategies described by participants, to group related strategies into thematic areas, and to map these strategies to published implementation strategies from the Expert Recommendations for Implementing Change (ERIC) taxonomy.(3) A second matrix was used to visualize participant data related to CFIR domains for implementation determinants across the eight interviews.

**References**

1. Jun GT, Ward J, Morris Z, Clarkson J. Health care process modelling: which method when? International Journal for Quality in Health Care. 2009;21(3):214–24.

2. Damschroder LJ, Aron DC, Keith RE, Kirsh SR, Alexander JA, Lowery JC. Fostering implementation of health services research findings into practice: a consolidated framework for advancing implementation science. Implementation science. 2009;4(1):1–15.

3. Powell BJ, Waltz TJ, Chinman MJ, Damschroder LJ, Smith JL, Matthieu MM, et al. A refined compilation of implementation strategies: results from the Expert Recommendations for Implementing Change (ERIC) project. Implementation science. 2015;10(1):1–14.

**Process Mapping Focus Group Facilitation Guide**

**Project Title: Implementation of low-threshold PrEP services in sexual wellness clinics in** Philadelphia

**Process Mapping Focus Group:** PrEP Stakeholders (PrEP Navigators, PrEP providers, benefits navigators, outreach specialists, social workers, nurses, front desk staff)

**Preparation:**

- Request a list of potential participants and their email addresses from the agency director or point person. Request that agency director or other agency leader send an email invitation to all potential participants with details about the activity, it’s purpose, and a brief agenda. A member of the research team should be copied on this email and their contact information made available to answer questions. Ask for an RSVP to get a sense of group size and how much food to bring.
- Identify a location in the agency that would be suitable for this group. It should be quiet and somewhat private, with enough chairs and tables for everyone, and some space on a wall to tape up the process map.
  - if you are going to use a table ensure chairs are spaced equally without any single individual sitting at the “head” or in a position of perceived authority
- Make a plan to get food for lunch.
- Gather needed materials
  - Large paper
  - Sticky notes in at least three colors
  - Sharpies
  - Tape
  - Charged phone battery for recording
  - Two team members (one facilitator, one note-taker/recorder).

**Introduction (15 minutes)**

- Gather everyone in the room and make sure everyone can get some food and get a seat. Ask everyone to fill out an informed consent form as they get settled. Explain that you would like to record the conversation so that no ideas are missed. (5 minutes)
- Short introduction: (2 minutes)
  - What is the purpose of this session?
    - PrEP can be a great option for HIV prevention for many people, but in Philadelphia there are still gaps in people’s access to PrEP, including racial disparities in who has access to PrEP. The health department estimates that about 14,000 Philadelphians could benefit from PrEP, but only about 3,000 have actually gotten connected to PrEP. The work of agencies like yours is a critical piece of the puzzle in addressing these gaps. By studying and learning from the successes and challenges that you all encounter in your daily work with PrEP and sexual health, we are hoping to work together to strengthen our ability to provide these important services, both here in your agency and in other agencies around the city.
  - What are we hoping to achieve?
    - We want to use an activity called Process Mapping that will allow us to visualize the system that your agency has in place for getting clients connected to PrEP. Then by reflecting on the parts of this system that are working well, and on parts that could use support or additional investment, we can start to paint a picture of the different strategies that might be most effective for serving the needs of our communities.
  - What will happen afterwards
    - During today's activity, we will draw out the steps involved in providing PrEP services at your agency. This map will highlight the strengths of your agency and will show the flow from start to finish. We will compile all the ideas generated from our discussion and share back with you a visual diagram of the process of delivering PrEP services at your agency, which we hope will be of use to you and your team. We will also share back with you at the end of the year some over-arching lessons learned across the whole project, which we hope will also help to inform the important work you all do for our community.
- Make sure everybody introduces themselves, one by one, this should be short and sweet (3 minutes)
- Set the ground rules for the day (1 minute)
  - Use only a first name or an alias when talking about specific individuals. These will be edited out in the transcripts from the recording.
  - Say if you don’t agree or don’t understand
  - Everyone has an equal voice
  - Ask participants to provide any additional ground rules that should be added
- Explain how the process map works and how we will build it together (2 minutes)
  - Process Mapping is a technique to help visualize the way that a group of people accomplish a goal. To do this, we will first define what the process is that we want to map by putting a Starting Point and an Ending Point on our map.
  - Next, we will brainstorm the various steps that take you from point A to point B. This may include tasks that are completed by different people or departments, so we will try to keep track of who is doing what by using the “lanes” here on the map.
  - As we talk, we may encounter parts of the process that you believe are working really well. In order to highlight those, we will add a Gold Sticky note to mark them. We may also find steps in the process that are challenging or not working as well as you think they should. We will mark these with a Blue Sticky note. Finally, we will also have an Idea List, where we can add ideas for improvement or change, and a Car Park, where we can put issues or ideas that need more discussion later (just so that they don’t slow down our mapping process).
- Are there any questions before we start? (3 minutes)

**Process Mapping (30 minutes)**

***Start recording here***

- Identify starting and ending points. This will be different for each agency, and the participants should identify where the starting and ending points are for their particular agency.
  - Here are a couple of possible examples
    - Start: Contact with client who is a potential PrEP candidate
    - End: Client receives 30 day PrEP supply OR client receives PrEP prescription OR client schedules PrEP appointment with outside agency OR client receives PrEP referral to outside agency.
  - Start identifying the steps that are taken to get from Start to End
  - Place stickies for each step in a lane labeled for the person or department that usually completes that step
  - Continue until you have reached the Ending Point.
  - Here are a few tips and reminders for facilitating this activity
    - Focus on what is happening in reality 80% of the time, NOT what should be happening or what the protocol says
    - Don’t forget to use the Car Park list if there is an issue or disagreement that cannot be resolved. If discussion lasts >5 mins on a particular step without resolution, consider using this to park the issue and move on
    - If ideas for improvement arise before you finish the map, consider using an “Idea List” to go back to when the map has been completed. This avoids losing that concept without the risk of being sidetracked
    - If you’re facilitating, AVOID the temptation to evaluate ideas as they’re generated as good/bad/ugly as this may discourage others to contribute
    - Make sure every idea is written on a sticky note and added to the list

**Discussion (15 minutes)**

- Looking at the map that we have created, and thinking about the goal of providing easy and smooth access to PrEP for your clients, I’d like to ask you a few questions about the process overall
  - What aspects of the process are working really well?
  - What are the special strengths and assets of your agency that support access to PrEP for the community?
  - Where are there sticking points or challenge points in the process?
  - What resources would help you address these challenges?
  - What goals do you have for the coming year around PrEP delivery?
  - What other ideas do you want recorded on the map?
- Wrap up and thank you. Let them know we would like to email them with the compiled map and ideas. Make sure everyone has your email and contact information in case they want to get in touch.

*Notes on modifications to this plan if in-person focus groups are not possible and if a virtual focus group will need to be conducted instead:*

- *Consent forms will be emailed to participants ahead of time so they can read and sign.*
- *Zoom will be used to hold the meeting.*
- *Meeting will be recorded, but only audio the file will be downloaded and saved. The video file will be deleted.*

- *<https://app.diagrams.net/> will be used to create the Process Map. Facilitator or assistant will share screen so that participants can see the progress.*

**Deidentified Example of Process Map**
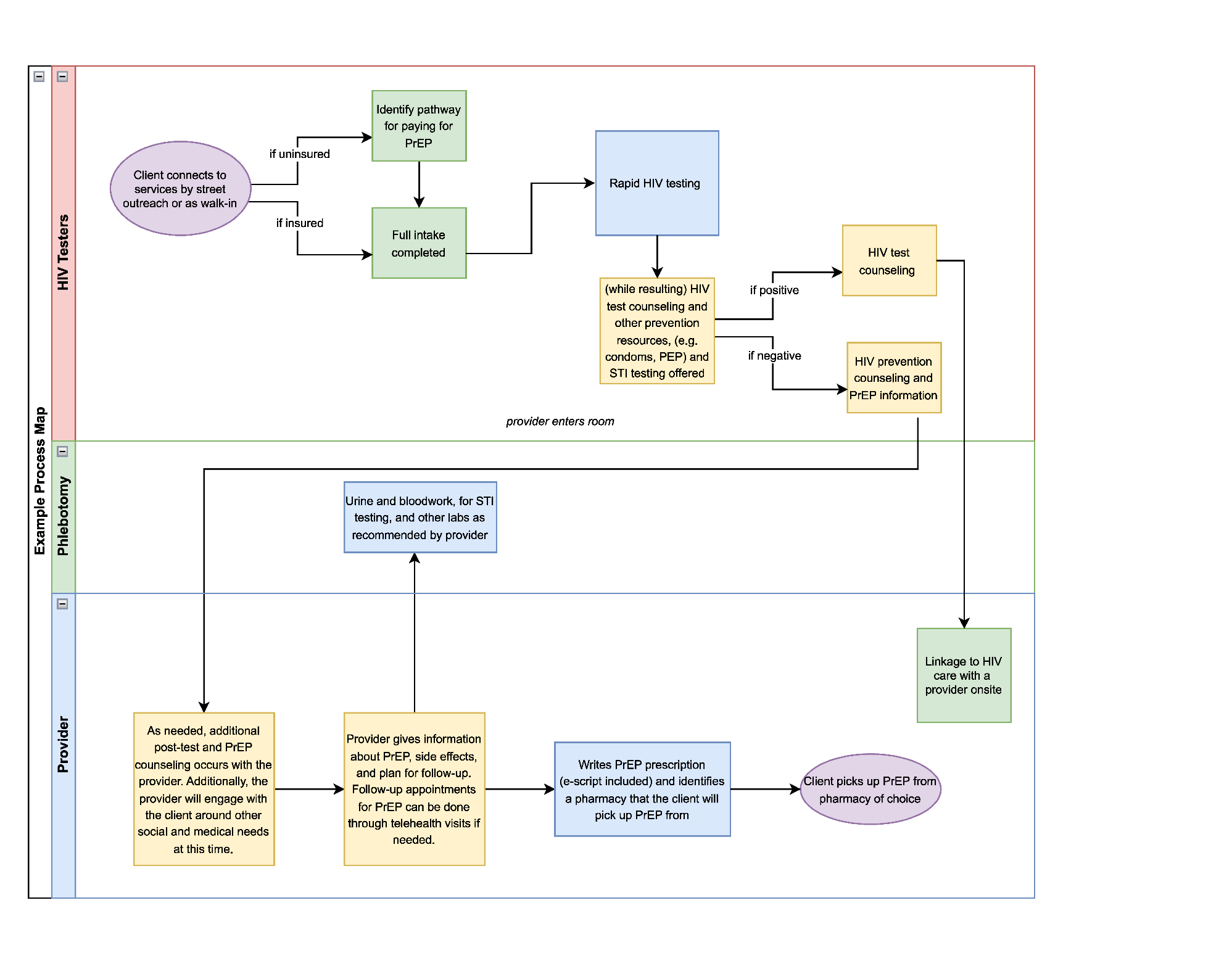


**Interview Guide**

Project Title: Implementation of low-threshold PrEP services in sexual wellness clinics in Philadelphia

*Before we start, I want to thank you again for your participation in this study. Your participation may contribute to a better understanding of strategies that can be used by community-based organizations to provide PrEP services that are easy to access and meet the needs of the communities they serve. You participated in a focus group earlier this year that aimed to describe the process of PrEP service delivery at your agency. In our conversation today, I’m hoping to ask some more detailed questions about how the process of delivering PrEP services has gone for you and your agency this year. I’d like to talk specifically about what strategies or systems have been most successful, and discuss where additional resources may be needed to optimize PrEP service delivery.*

*I want to remind you that everything we talk about today will be completely confidential. You may speak freely about your thoughts, opinions, and experiences. We will be recording this interview; however, the audio file will only be used by the research team (comprised of myself and one research assistant) and a professional transcription agency. To protect your privacy and that of others you may mention, I will not ask identifying questions about the people or organizations you talk about.*

*I expect this interview to run between 45 minutes to an hour and we can take a break if needed. Remember, if you ever feel uncomfortable or do not want to answer any of these questions, we can pause or stop the interview at any time. Before we begin, do you have any questions?*

*[Space for questions]*

*****

*[Begin recording]*

- My first question is an easy one: what is your role at [name of agency]?

*Great thanks! I want to ask some questions about the specific strategies being used to make PrEP services easy to access at your agency*

1. Reflection on Implementation Strategies
   - 1. What other key strategies has your agency used to make PrEP easy to access?

*Thinking about each of these strategies that your agency uses to make PrEP easy to access, I want to ask some questions about each individual strategy.*

1. Characteristics of Intervention (asked for each strategy)
   1. Relative Advantage
      1. How does this strategy for making PrEP easy to access compare with previous strategies or alternative strategies?
   2. Adaptability
      1. Are there components of this strategy that should be altered?
      2. Are there components of this strategy that should NOT be altered?

*Okay next I’d like to reflect on the whole process of providing PrEP services by looking at the process map that we drew earlier this year during the focus group. This map is meant to visualize the whole process of connecting clients to PrEP, from outreach, to HIV testing, to PrEP counseling, to making a referral or scheduling an appointment with a PrEP provider, to the client picking up their medication.*

*[Share screen to show process map]*

1. Process
   1. Executing
      1. Looking at this map and considering how PrEP services are currently provided at your agency, is this still an accurate picture of the process?
      2. What changes, modifications, or adaptations have been made to your PrEP service delivery since July 2021 (if any)? When did those changes happen?

[Stop sharing screen]

- 1. Reflecting and Evaluating
     1. How do you measure progress towards goals for PrEP services?
        1. Do staff get any feedback reports about their work towards these goals?
        2. Who is involved in setting these goals?

1. Outer Setting
   1. Client Needs and Resources
      1. How well do you think the PrEP delivery strategies that have been used this year at your agency are meeting the needs of the individuals served by your organization?
         1. In what ways will the intervention meet their needs? E.g. improved access to services? Reduced wait times? Reduced travel time and expense?
   2. External Policies and Incentives
      1. What kind of financial or other incentives from outside your agency influenced how PrEP services were provided in your agency?

Next, I’d like to ask you about how PrEP service delivery fits into your agency as a whole.

1. Inner Setting
   1. Culture
      1. How do you think your organization's culture (general beliefs, values, assumptions that people embrace) affect the implementation of these PrEP delivery strategies?
   2. Implementation Climate
      1. To what extent might the implementation of these PrEP delivery strategies take a backseat to other high-priority initiatives going on now?
   3. Readiness for Implementation
      1. Do you expect to have sufficient resources to implement and administer these PrEP delivery strategies? Where will these resources come from?

Okay, thank you for all this very insightful information. For this last section, I always just ask a few final questions about the interview.

1. Conclusion
   1. Is there anything that I haven’t asked you that you think I should have?
   2. Anything that you feel is missing or should have been asked about in a different way?
   3. Any other questions or comments?

Okay, that concludes the interview.

[stop recording]
