# Supplementary material for: Advancing the community plan to end the HIV Epidemic in Philadelphia: a qualitative descriptive evaluation of low-threshold PrEP services in sexual health clinics
Source: Implement Sci Commun. 2024 Jan 5;5:4. doi: 10.1186/s43058-023-00543-y (PMC10768374; doi:10.1186/s43058-023-00543-y)
Supplement: Supplementary file 3 — Additional file 3: Supplemental file 3. Additional Results Details. [file 43058_2023_543_MOESM3_ESM.docx]

**Additional Results Details**

**Focus Group Process Maps**

The process of PrEP delivery described by focus group participants aligned with the four phases outlined in the city’s guidance on low-threshold PrEP delivery. Regarding connection to services, most clients were connected through community-based outreach programs (e.g., health fairs, community-based HIV testing) or as walk-ins at the clinic site. Clinics all conducted a basic intake with clients before completing HIV testing; for clients interested in PrEP, a more comprehensive intake was completed after HIV testing and before visiting with a PrEP provider. Insurance information was typically collected before HIV testing to assess specific needs that would inform patient navigation services. Patients who were uninsured were connected to insurance or medical assistance programs if eligible, however all clinics were able to provide PrEP services to patients regardless of insurance status.

During the prevention navigation phase, prevention navigators performed rapid HIV testing and offered additional prevention resources (e.g. condoms, linkage to nPEP) while waiting for test results. One clinic described using an opt-out strategy for HIV testing that aimed to increase testing uptake while minimizing HIV stigma. At all clinics, the prevention navigator would coordinate a warm handoff to an onsite HIV care provider for any client testing positive for HIV. For clients with a non-reactive HIV result, information and education about PrEP was provided by prevention navigators. Clients interested in PrEP were then scheduled for an appointment with a PrEP provider of their choosing, with the option to schedule with a provider on site. Two clinics reported that clients could typically be scheduled for a same-day appointment and one clinic described sometimes scheduling for same-day appointments, but typically scheduling within two days of the initial encounter.

The clinical encounter was conducted by a licensed physician, nurse practitioner, or physician’s assistant. This visit included an assessment of medical history, social history, sexual health and behaviors, and patient goals. One clinic described occasionally conducting provider visits for PrEP over telehealth, although this was not a common practice. At all clinics, an onsite medical assistant or other trained professional conducted phlebotomy for lab-based HIV testing, STI testing, and other lab. Clinics described some variation in when PrEP prescriptions were written, with two clinics providing prescriptions and a seven-day sample of PrEP on the day of the appointment, and one clinic reporting waiting to write the prescription until lab results were returned.

Prevention navigators or clinical staff performed follow-up, which consisted of ensuring prescriptions were sent to a pharmacy accessible to the patient and scheduling follow-up appointments at three months intervals. One clinic had a pharmacy onsite which facilitated rapid access to medication, and another clinic offered telehealth visits for follow-up visits.

***Implementation Determinants from In-depth Interviews***

Themes from the in-depth interviews around implementation determinants emerged within the CFIR domains of Inner Setting, Outer Setting, and Process. No themes arose in the domains of Intervention Characteristics or Characteristics of Individuals.

**Inner Setting.** Most participants discussed their organizational culture as open and welcoming to all people, regardless of background or identity; however, several participants noted that staff training and other efforts to improve equity and inclusion in the workplace were ongoing and additional work was needed to realize this vision of a welcoming environment for all. Additionally, an organizational commitment to centering services on client needs and priorities was a common theme. Participants described how flexibility in scheduling and centering client choice were key facilitators to delivery low threshold sexual health services. One participant reported that hiring exclusively bilingual staff was a major strength in providing accessible services to Spanish-speaking clients at their clinic.

Participants varied in the degree to which they felt PrEP implementation was a high priority at their clinic. All participants reported that PrEP implementation was among their clinic’s priorities, but several recognized that competing priorities at the clinic level (e.g., meeting other grant requirements) could lower the relative priority of PrEP implementation. Participants described staff shortages as a challenge that limited community-based outreach and sometimes resulted in delays in scheduling an appointment with a PrEP provider.

**Outer Setting.** Participants reported that clients often have high-priority social needs beyond sexual health, including homelessness and food insecurity, that influenced how PrEP services were implemented and often created challenges to retaining clients in care. Transportation and scheduling barriers were also common concerns. Participants reported that expanded hours and providing assistance with transportation costs could help overcome these barriers. Providing financial incentives, such as a small gift card, for engaging with various components of the PrEP process also helped to balance prevention with other priorities. Additionally, guidance and grant requirements from the city and other funders were key drivers of service priorities and shaped the service delivery models. Resources from the city, including funding for staffing, training and education, and literature and other materials have supported the implementation of these low-threshold PrEP service models.

**Process.** During the study period, adaptations to the PrEP service delivery model were made to increase efficiency and lower barriers to accessing PrEP. Several participants reported changes to the workflow that incorporated additional points for conversations about PrEP, from intake, to testing, to patient navigation. Modifications to the clinical workflow also helped improve efficiency, including streamlining specimen collection for STI tests and other lab work. Goals for PrEP service delivery benchmarks were generally set by department supervisors and clinic leadership, and typically reflected measures that were required for reporting to funders and as well as measures reflecting clinic priorities. In most clinics, goals for PrEP benchmarks were discussed at staff meetings on a monthly basis with additional informal discussions between staff and leadership on a more frequent basis.
